# Inductively actuated micro needles for on-demand intracellular delivery

**DOI:** 10.1038/s41598-018-28194-3

**Published:** 2018-07-02

**Authors:** Mincho N. Kavaldzhiev, Jose E. Perez, Rachid Sougrat, Ptissam Bergam, Timothy Ravasi, Jürgen Kosel

**Affiliations:** 1 Computer, Electrical and Mathematical Science and Engineering, King Abdullah University of Science and Technology, Thuwal, 23955-6900 Saudi Arabia; 2 Biological and Environmental Sciences and Engineering, King Abdullah University of Science and Technology, Thuwal, 23955-6900 Saudi Arabia; 3 Imaging and Characterization Core Lab-EM, King Abdullah University of Science and Technology, Thuwal, 23955-6900 Saudi Arabia

## Abstract

Methods that provide controlled influx of molecules into cells are of critical importance for uncovering cellular mechanisms, drug development and synthetic biology. However, reliable intracellular delivery without adversely affecting the cells is a major challenge. We developed a platform for on-demand intracellular delivery applications, with which cell membrane penetration is achieved by inductive heating of micro needles. The micro needles of around 1 μm in diameter and 5 μm in length are made of gold using a silicon-based micro fabrication process that provides flexibility with respect to the needles’ dimensions, pitch, shell thickness and the covered area. Experiments with HCT 116 colon cancer cells showed a high biocompatibility of the gold needle platform. Transmission electron microscopy of the cell-needle interface revealed folding of the cell membrane around the needle without penetration, preventing any delivery, which was confirmed using the EthD-1 fluorescent dye. The application of an alternating magnetic field, however, resulted in the delivery of EthD-1 by localized heating of the micro needles. Fluorescence quantification showed that intracellular delivery, with as high as 75% efficiency, is achieved for specific treatment times between 1 and 5 minutes. Overexposure of the cells to the heated micro needles, i.e. longer magnetic field application, leads to an increase in cell death, which can be exploited for cleaning the platform. This method allows to perform intracellular deliver by remotely activating the micro needles via a magnetic field, and it is controlled by the application time, making it a versatile and easy to use method. The wireless actuation could also be an attractive feature for *in-vivo* delivery and implantable devices.

## Introduction

Biologically active molecules are heavily used in therapeutics, such as proteins and peptides^1,2^ or RNAs to modulate gene expression^3,4^. Most of these molecules are not able to permeate the cell membrane, due to their size, surface charge or instability and thus require external means to be transported into the cell cytoplasm and to be functional^5,6^. Several approaches have been developed for intracellular delivery, which include viral vectors^7^, cell-penetrating peptides and proteins^8–10^, microneedles^11^ or nanoparticles^12–14^ as carriers, electroporation^15–18^ and carbon nanotubes coupled to Atomic Force Microscopy^19^. These methods, however, have limitations in the form of cell toxicity^20^, cell type specificity^21^, mutagenesis, undesired immune responses^22^, questionable transfection^23^, targeting^24^ and *in vivo* applicability^25^.

An ideal intracellular delivery system should provide high biocompatibility, be applicable to a wide range of cell types and be scalable to high-throughput studies^26^. In addition, tight control over the delivered dose and timing are essential for understanding the cellular response. Although the mechanism of delivery using needles is still debated and is probably time^27^ and surface-dependent^28^, successful and efficient intracellular delivery has been reported for cells growing on silicon nanowires^26^ and diamond nanoneedle arrays^29–31^. Similarly, in one of the only *in vivo* approaches, silicon nanoneedles were used to deliver nucleic acids and modulate gene expression to induce angiogenesis and increase blood perfusion with a very high efficiency in mice^32,33^.

High aspect ratio microneedles have been previously reported as a suitable platform for cell transfection with high efficiencies^34,35^, but offering limited transfection control by their mechanical, passive delivery mechanism. Moreover, such systems are not cell specific, making them impractical for heterogeneous cell cultures.

Here, we report intracellular delivery using arrays of gold micro needles, which are wirelessly controlled to penetrate the cell membrane on-demand by induced localized heating. Control over the delivery is achieved by manipulating the timing and duration of the heat induction. The magnetically induced heating provides a high transfection control and allows monitoring of the cell culture during delivery. It can potentially be expanded to *in-vivo* applications, since the applied magnetic fields are in the range of those used for magnetic particle hyperthermia^36,37^. The micro needles are fabricated by electrodeposition into an amorphous Si template, a process that is highly reproducible and can easily be scaled up. The micro needles’ length, diameter and spacing can be modulated through the fabrication process in order to provide adequate interfacing with different kinds of cells.

## Results and Discussions

### Amorphous Silicon Template

The micro needles are fabricated by electrodeposition of gold into amorphous silicon (ASi) templates. The ASi is grown on top of a Si wafer with a SiO_2_ insulation layer and a gold seed layer for electrodeposition. Patterning of the ASi is achieved using a chromium (Cr) hard mask and an etching process, resulting in a ASi template with micron-sized holes and a gold electrode at its bottom.

**Figure 1:**
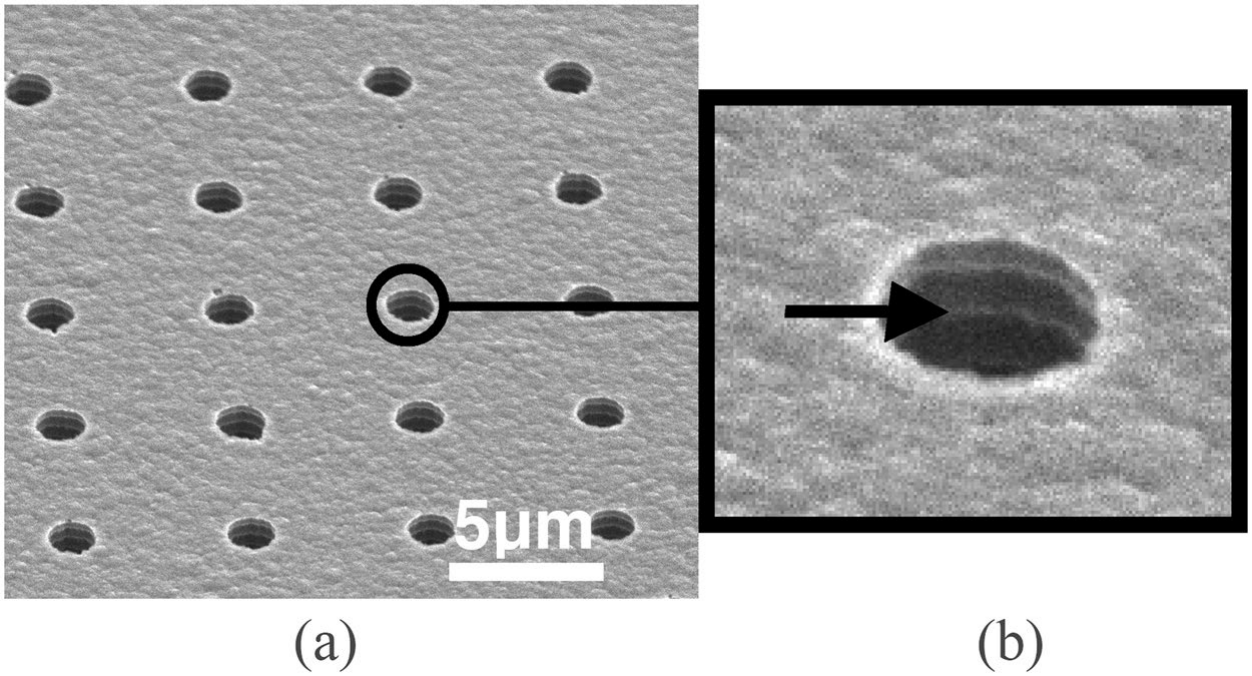
(**a**) SEM image of an amorphous Si template with an array of 36000 holes of 1.5 μm diameter and 5 μm depth. (**b**) Close-up views on the template holes.

Figure 1(a) shows an SEM image (top view) of holes generated in the ASi by deep reactive ion etching (DRIE, Bosch process)^38^. The holes are 1.5 μm in diameter with 5 μm spacing (edge to edge) and 5 μm in depth. A close-up view is given in Fig. 1(b).

### Gold micro needles

**Figure 2:**
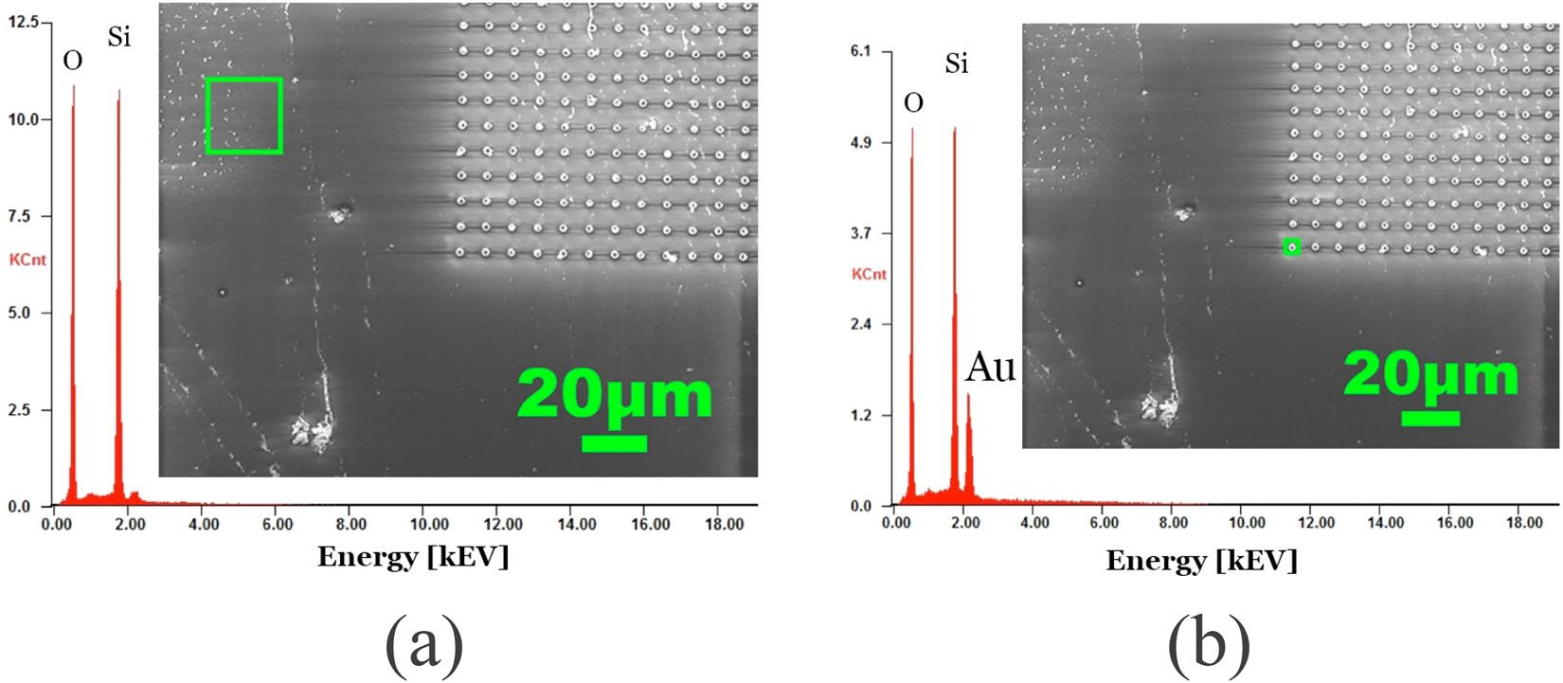
EDX analysis of the silicon substrate with electroplated gold needles. (**a**) EDX of an area (green rectangle) without needles showing the signal from the substrate and revealing no remaining contamination of carbon, the gold electrode or amorphous silicon. (**b**) EDX of the needles showing a distinct peak of gold for the needles and the substrate signals.

After electrodeposition of the gold micro needles (see supplementary information and Fig. S1) and removal of the ASi template, energy dispersive x-ray spectroscopy (EDX) analysis shows that the substrate surface is composed of Si and O_2_ atoms outside of the micro needle area (Fig. 2(a)). On the other hand, EDX analysis of needles with 1.5 μm diameter and 5 μm height shows a strong signal of gold together with Si and O_2_, whereby the latter two account for the substrate surface (Fig. 2(b)). These results show that all the reactive elements from the etching process are evacuated and not re-deposited on the needles or on the surface.

**Figure 3:**
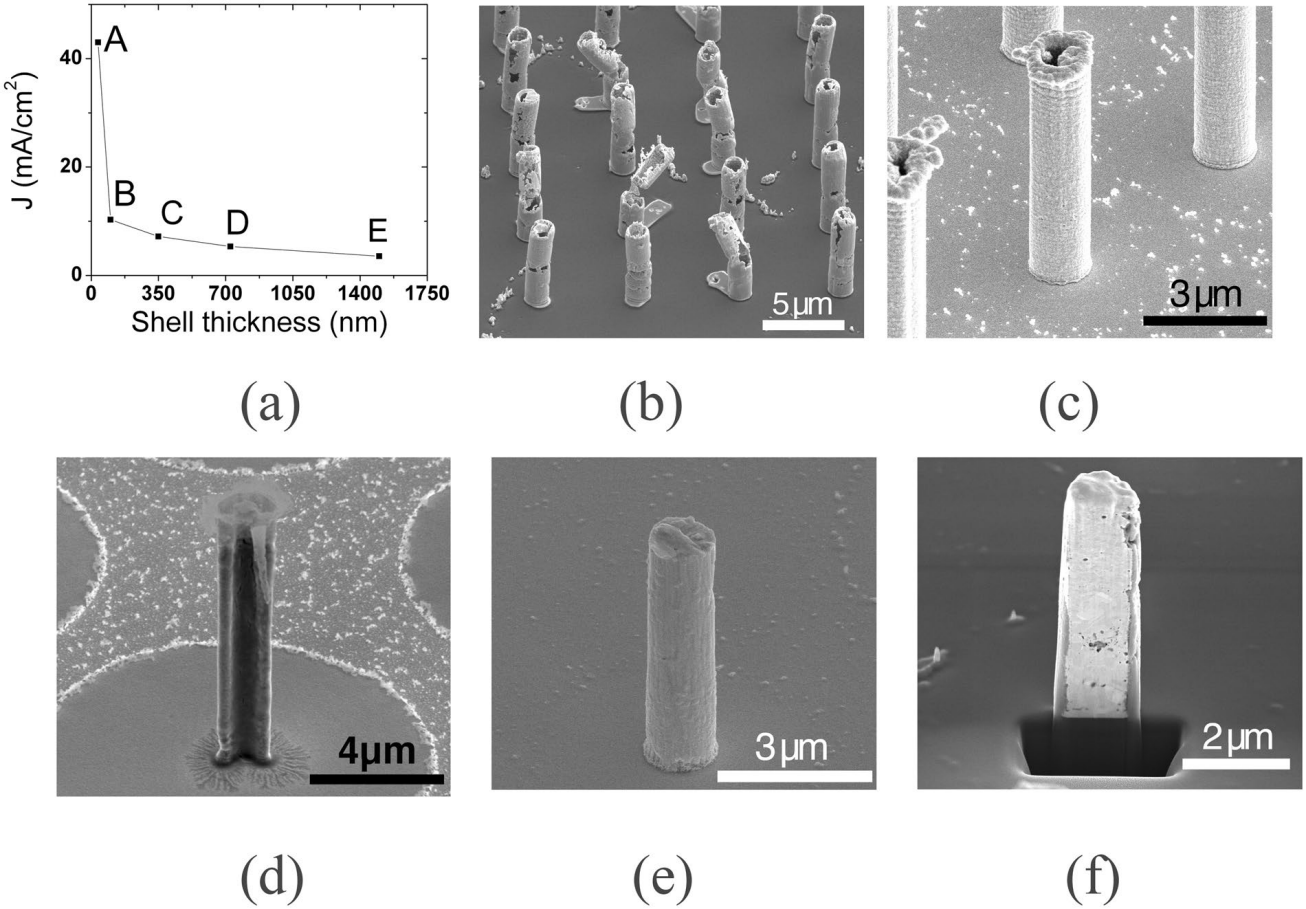
(**a**) Dependence of the needle structure on the current density (J). (**b**) SEM image of gold needles electrodeposited with a high current density (**a** point A). (**c**) SEM image of gold shell needle and its cross section (**d**) deposited with medium current densities (**a** points B and C). (**e**) SEM image of gold filled needle and its cross section (**f**) deposited with a low current density (**a** points D and E).

Specific needle structures can be obtained by tailoring the electroplating current densities, which was previously also reported^39^. The structure of needles deposited into 1.5 μm wide pores ranges from shells with 200 nm wall thickness to filled needles with 1.5 μm in diameter as shown in Fig. 3(a). At large current densities from 50 to 20 mA/cm^2^ (Fig. 3(a), points from A to B) the deposition results in gold clusters that agglomerate along the edges of the ASi template, and the final needle structures are rather fragile (Fig. 3(b)). Needles produced within the range of 10 mA/cm^2^–4 mA/cm^2^ (Fig. 3(a), points B to D) have a solid shell structure (Fig. 3(c)) and a homogenous wall thickness of 300 nm, as shown in the cross section image in Fig. 3(d). Decreasing the current density to <4 mA/cm^2^ produces filled gold needles, shown in Fig. 3(e), with their cross section shown in Fig. 3(f). As seen from Fig. 3(d,f), there are no remainders of the gold electrode layer outside of the needle area, which confirms the EDX results from Fig. 2(a).

### Cell viability and growth on gold micro needles

**Figure 4:**
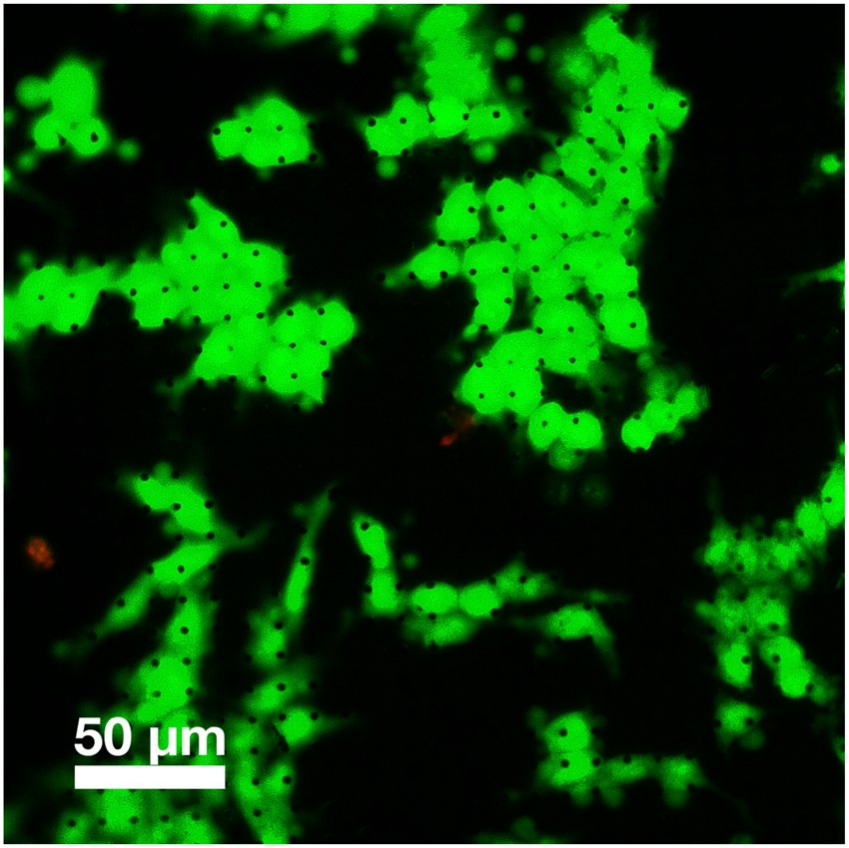
Fluorescence microscopy of calcein/EthD-1 stained HCT 116 cells grown on 5 µm long needles (visible as black dots) with 1, 5 µm diameter and 10 µm pitch.

The cell viability on the micro needle substrates was assessed using the calcein AM and ethidium homodimer-1 (EthD-1) fluorescence staining method. Figure 4 shows that most of the cells on the micro needles are calcein-stained 24 hours after growth, indicating a high biocompatibility. Similar results have been observed for other micro needle systems used for the same purpose^35^.

**Figure 5:**
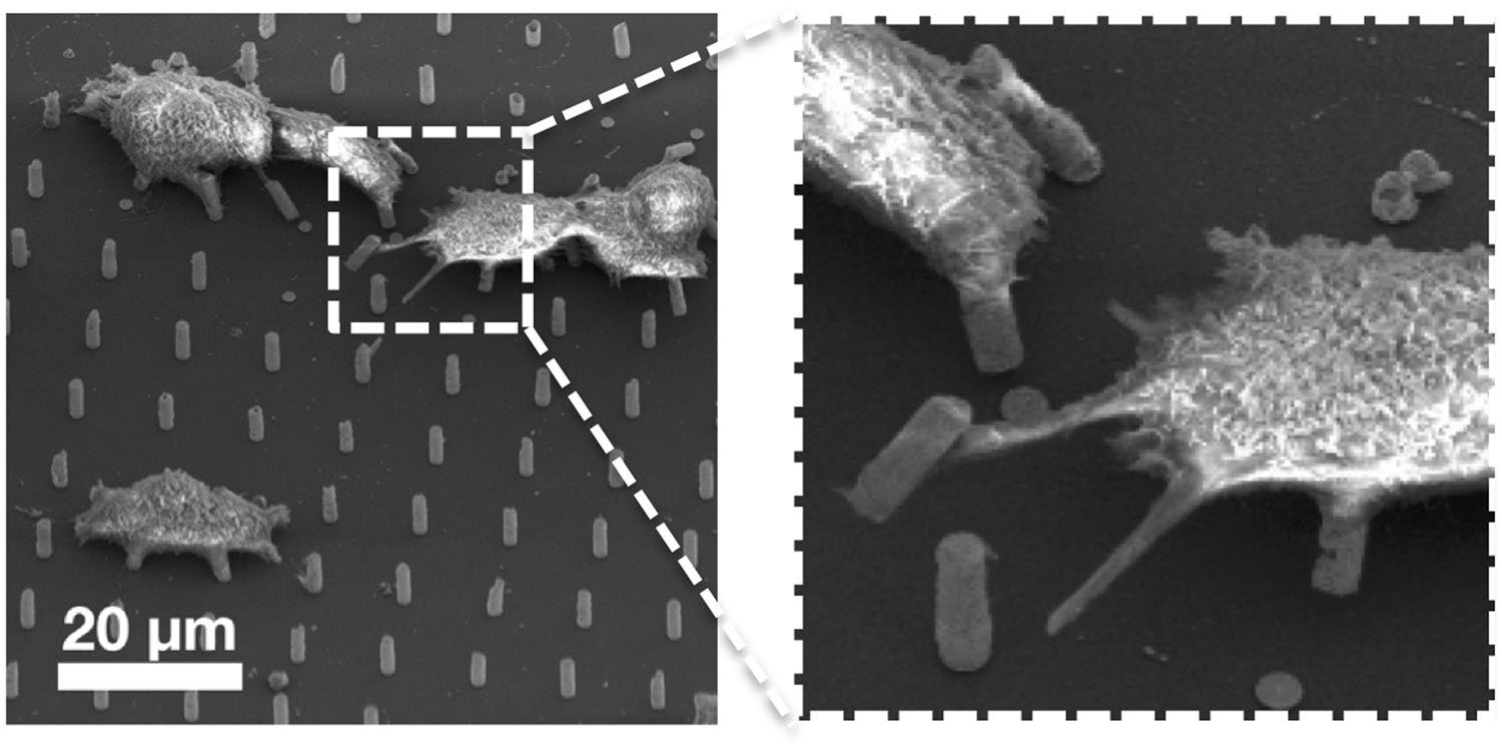
SEM image of HCT116 cells on 5 µm long needles with a diameter of 1.5 µm. Cells show a conventional morphology with focal adhesion points on the pillars (inset).

**Figure 6:**
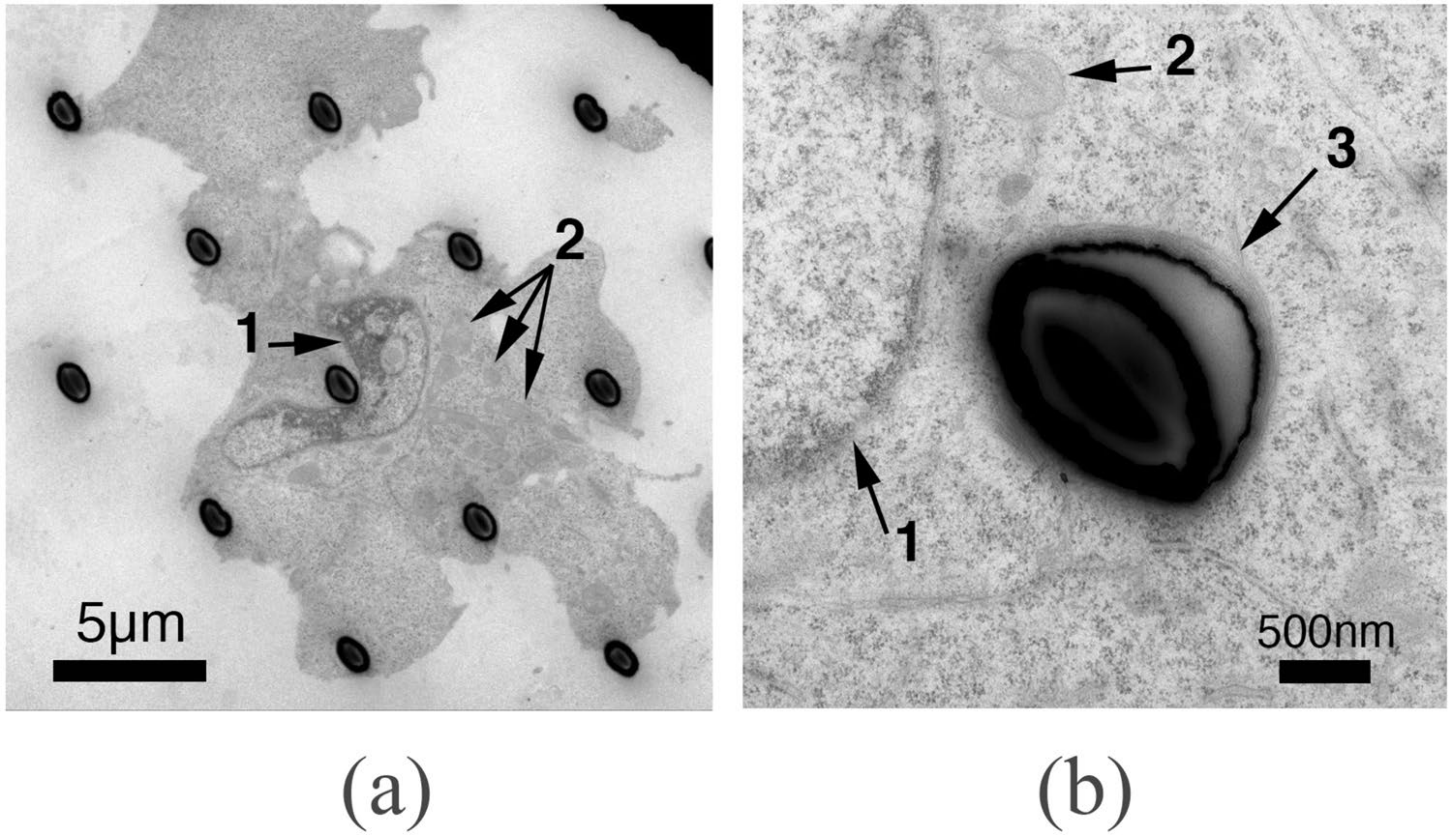
Transmission electron microscopy images of HCT116 cells on micro needles. (**a**) Cells grown on 5 µm long needles. The nucleus (arrow 1) is bent around a needle. The structure of the mitochondria (arrow 2) and endoplasmic reticulum are well maintained and similar to the one of healthy cells. (**b**) A filament layer surrounds the periphery of the interface with the needle (arrow 3).

Electron microscopy was employed in order to study the interface between the cells and the micro needles. From Fig. 5 it can be seen that HCT 116 cells wrap around the needles as they form adhesion points, ultimately settling on multiple structures. This scenario has been proposed as an outcome that depends on the distance between the needles^28^. A closer look reveals that the cells adapt their cytoskeleton, elongating it, in order to reach for farther micro needles, and in some cases the needles are bent in the direction of the cells, suggesting the cells were using them as anchors and applying a significant force to them. A closer look, obtained by transmission electron microscopy (Fig. 6), indicates that the cells manage to spread around the needles and preserve their integrity. In the case shown in Fig. 6(a), the nucleus (arrow 1) folds around a micro needle and the intracellular structures are well maintained. A structure (arrow 3 in Fig. 6(b)) that could be composed of actin filaments can be observed around the periphery of the interface.

It should be noted that, during the ultramicrotomy preparation for TEM (Fig. 6(a,b)) the needles have been compressed to an oval shape, an artifact from the diamond knife cutting, which is stressing the micro needles, as previously reported^40,41^.

### Intracellular delivery

**Figure 7:**
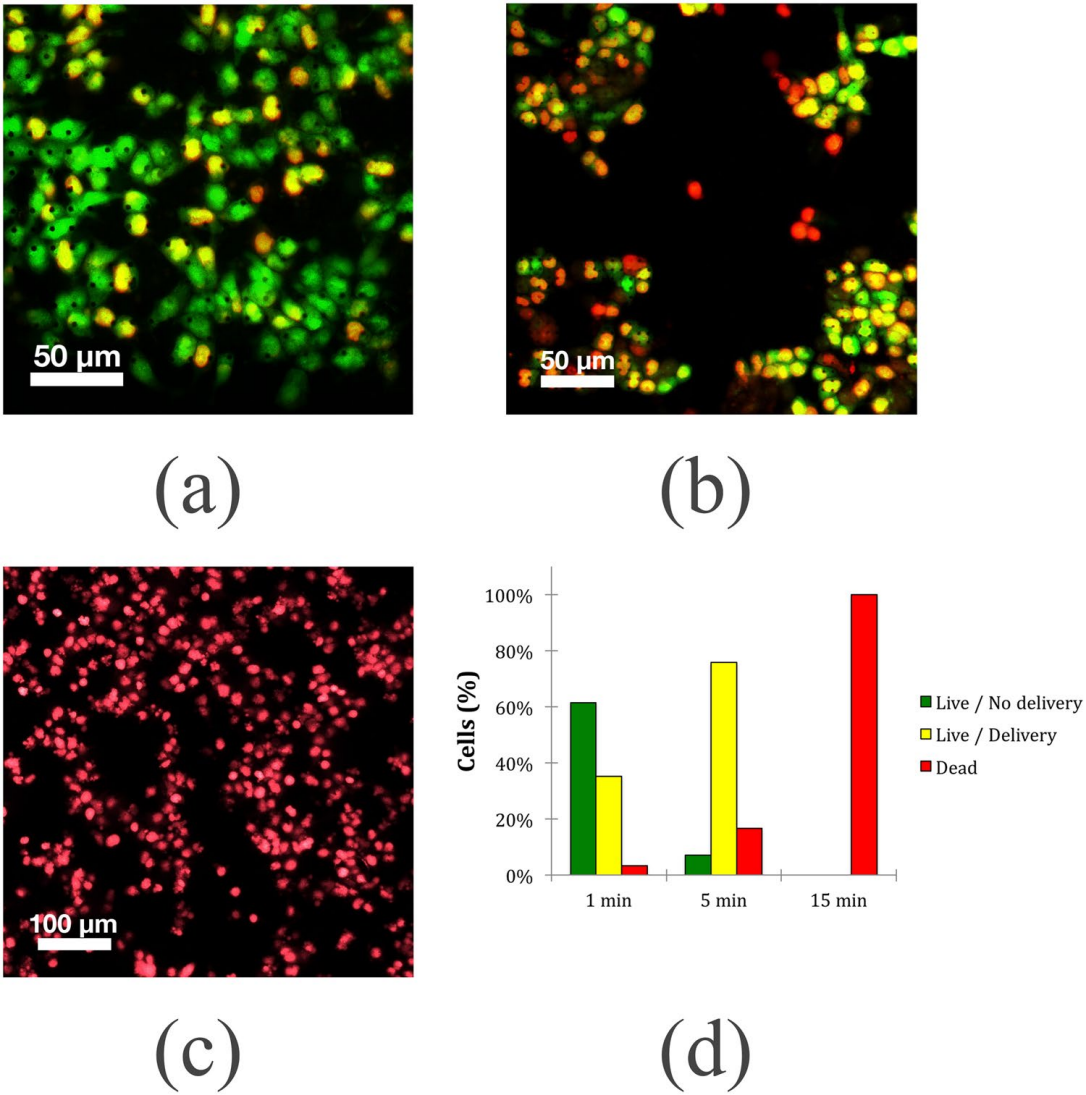
Fluorescence imaging of calcein AM and EthD-1 stained HCT 116 cells after application of a magnetic field of 360 Oe and 425 kHz for three different times. (**a**) 1 minute. (**b**) 5 minutes. (**c**) 15 minutes. For each time point, three independent groups of cells were grown for 24 hours and then stained and imaged. A treatment time of 1 minute and 5 minutes showed delivery efficiencies of 35% and 75%, respectively. Cell death of the entire population was observed for the group of cells placed under a field for 15 minutes.

The capabilities of the mirco needle substrate for intracellular delivery were studied by applying a field of 360 Oe and 425 kHz (see supplementary information Fig. S2). When applying the field for a duration of one minute, direct quantification of the number of cells yielded a delivery efficiency of 35%, evidenced by the co-localization of the calcein and ethidium homodimer-1 dyes, and a negligible amount of cell death (Fig. 7(a)). Applying the field for a time of five minutes increased delivery to approximately 75% of the cells. In this case, the percentage of dead cells was 17% (Fig. 7(b)). If the field is applied for 15 min, the whole cell population dies, as shown in Fig. 7(c). Figure 7(d) summarizes the obtained results with respect to the three staining conditions: live, dead and intracellular delivery. The results show that the efficiency of intracellular delivery is dependent on the time of exposure, and the latter also affects cell survivability. This indicates that the localized heating of the micro needles achieved by applying the magnetic field enables intracellular delivery. This process takes some time, as evidenced by the higher delivery rate after 5 minutes than after 1 minute. Eventually, if the field is applied for an extended period of time, an increase in temperature of the entire cell environment leads to cell death. The delivery efficiency observed here for the 1 minute treatment time is similar to that reported for carbon nanosyringes^42^ and diamond nano needles^29^, whereas treatment time of 5 minutes provides comparable delivery efficiency to that observed by Harding and colleagues^35^.

As shown in supplementary Fig. S3, the temperature of 1 ml solution heated by 43200 gold microneedles remains below 45 °C. Therefore, the stability of other cargos like nucleic acids or proteins should not be affected by this method^43–45^.

## Conclusion

We developed a gold micro needle platform with needles of 1.5 μm diameter and 5 μm height that enables on-demand intracellular delivery. The needles can be inductively heated by applying an alternating magnetic field, and the effect of heat application has been tested on the HCT 116 cell line. The fabrication of the micro needles utilizes cost-efficient standard microfabrication processes, encompassing electroplating of the needles into ASi templates. This process provides a high degree of customization of the needles’ dimensions and areal density, allowing fine-tuning for a large number of applications and accommodating multiple types of cell morphologies. The needle structure can be tailored by the current density during the electrodeposition. A high current density produces shell structures with increasingly thicker shells as the current density goes down, providing control over the diameter of the needles. Culturing HCT 116 cells on the developed platform revealed a high biocompatibility of the gold needles and SiO_2_ substrate. The micro needles are well tolerated by the cells, which grow on top of them and wrap around their tips. Using the calcein and EthD-1 dyes, no delivery was found when no magnetic field was applied. This is in line with the results of many previous studies that showed no cell membrane permeation when cells grow on top of micro or nano structures and no additional mechanism is used to force membrane penetration.

In case of the presented micro needle platform, this additional mechanism is wirelessly induced heating of the needles by a magnetic field. This method does not disturb existing routines or lab setups, since the coils can be conveniently placed underneath the cell culture substrate, and no additional wires or tubes need to be connected to the substrate. It also does not obscure the view on the cells during the field application, as is the case with methods that enable delivery by pushing needles into the cells from the top. We found that the duration of field application is a crucial parameter to control the delivery efficiency. If the field was applied for 1 min, intracellular delivery was achieved in 35% of the cell population, whereas a 5 minutes treatment time resulted in a delivery rate in 75% of the cell population with a small increase in cell death.

The wireless control enables simple operation without the need of a battery or cables connected to the device. This makes the method also attractive for *in-vivo* applications in combination with transdermal or implantable device concepts, considering that the magnetic field applied is in the range of the one used for magnetic particle hyperthermia.

## Experimental Section

### Amorphous-Silicon Template fabrication

**Figure 8:**
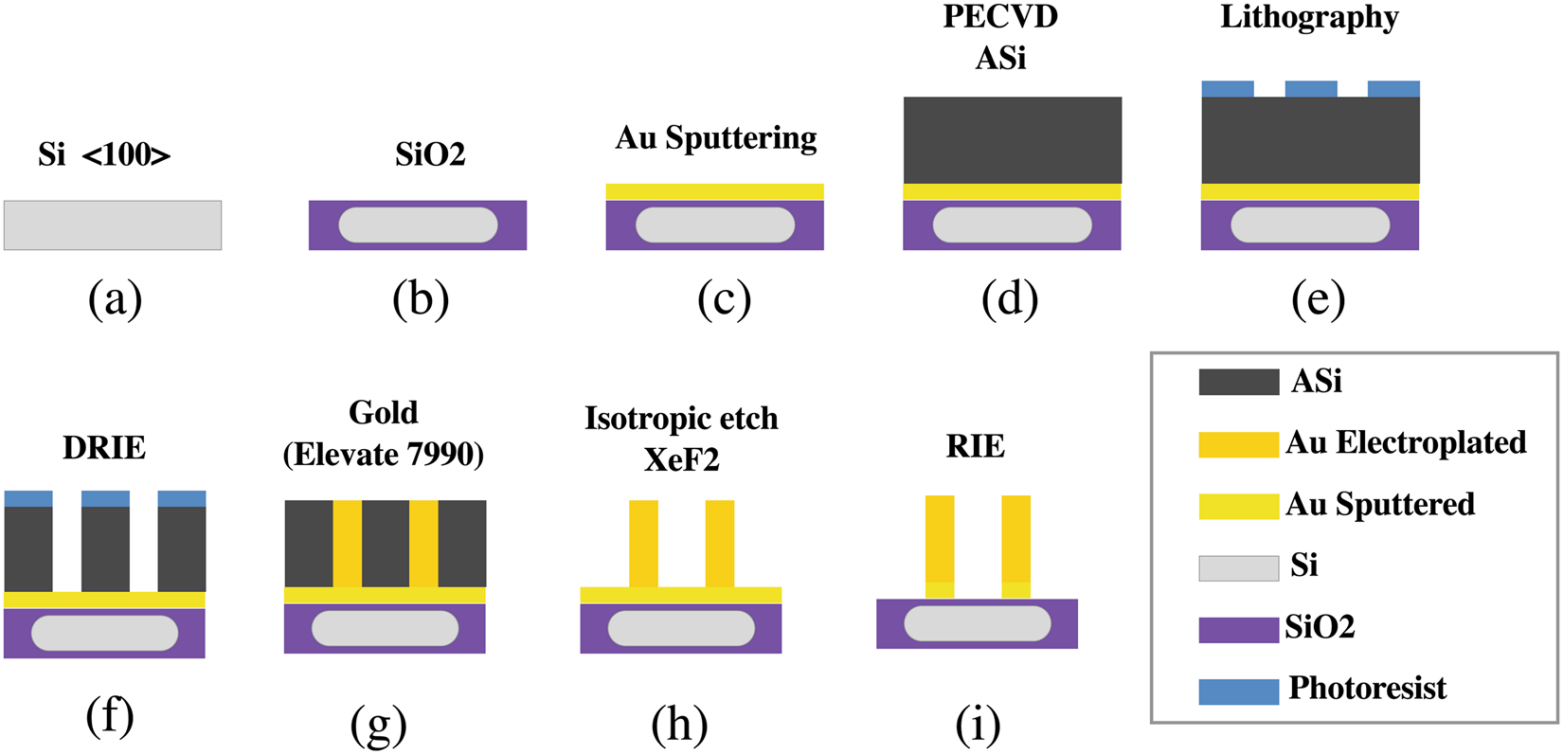
(**a**–**f**) Fabrication schematics of amorphous silicon template. (**g**–**i**) Electroplating of gold micro needles inside of the ASi template followed by template removal.

A 4′′ single-side polished Si wafer of 525 μm ± 25 μm thickness is used as substrate material (Fig. 8). The wafer is thermally oxidized to form a layer of 520 nm SiO_2_ (Fig. 8) in order to provide a non-conductive isolating surface. After that, 15 nm of Titanium and 100 nm of Gold are deposited by sputtering, forming the seeding electrode layer for electroplating. Plasma enhanced chemical vapor deposition is used to deposit 10 mm of amorphous-Si (ASi). The sample is initially preheated to 250 °C, followed by a cleaning step by plasma etching with NH_3_ at 40 sccm and N_2_ at 100 sccm, with RF power of 40 W at a pressure of 1000 mTorr, eliminating all dangling bonds on the surface and passivating it for ASi deposition. The deposition of ASi is effectuated by plasma composed of Silane (SiH_4_) of 25 sccm and Ar gas of 475 sccm, with a deposition rate of 0.2 mm/min. During deposition the pressure is maintained at 800 mTorr with RF power of 20 W as described in Table 1. Once the structural layer of ASi is deposited, the sample cools down for 10 min from 250 °C to room temperature. Cleaning of the sample before lithography is done in an isopropanol path preheated to 45 °C with sonication at 40 kHz for 2 min.

**Table 1:** Parameters for amorphous Silicon Deposition with Oxford PlasmaLab 100 Systems.

Parameter	Value	Gases	Value
Pressure	800 mTorr	SiH_4_	25 sccm
RF	20 W	Ar	475 sccm
Temperature	250 °C		

Next, the sample is heated to 90 °C for drying purposes and for a better adhesion with the resist. The negative photolithography resist MAN2403 is spin coated at a speed of 3000 rpm with a ramp of 1500 rpm/min for 30 sec onto the sample with the resulting thickness of 343 nm. The resist is baked to evacuate the solvent at 90 °C for 1 min. Then, patterning takes place with electron beam lithography (EBL, CRESTEC 9500C) at 50 keV with a writing current of 500 pA and a dose of 0.6 mC/cm. The development of the sample is performed in a 100 ml beaker with 30 ml of Dev-545 for 30 sec. A hard mask of 15 nm of chromium is deposited followed by the lift-off of the MAN2403 by acetone with 5 sec of sonication. The sample is cleaned with isopropanol for 20 sec and DI water for 1 min. To create the wells for the needles, the ASi is etched by deep reactive ion etching (PlasmaLab System 100, Oxford Instruments) with the parameters from Table 2, where cycles of wall passivation and bottom etching are repeated to obtain anisotropic etching. To create the holes, the template is subjected to 80 cycles of etching plasma. The selectivity of the etching plasma prevents the etching of the bottom gold electrode (100 nm) and opens a straight profile for electroplating contacting. During the etching process, a carbon deposition from the passivation step remains on the surface of the sample and reactive ion etching with O_2_ plasma (50 sccm O_2_, 50 mTorr, RF-200W, ICP-350 W) is applied for cleaning the surface for 15 min. Finally, oxygen plasma reactive ion etching (ICP 200 Watts, P = 10 mTorr) is applied for 2 min to remove the Chromium hard-mask layer.

**Table 2:** Parameters for amorphous Silicon Etching with DRIE Oxford PlasmaLab 100 Systems.

Parameter	Value	Gases	Passivation	Etching
Pressure	10 mTorr	C_4_F_8_	100 sccm	5 sccm
RF	35 W	SF_6_	5 sccm	100 scc
ICP	250 W	Ar	50 sccm	50 sccm
Temperature	10 °C			

### HCT 116 cell culture

HCT 116 colorectal carcinoma cells (ATCC® CCL-247) were grown in McCoy’s 5 A modified medium (ATCC®) supplemented with 10% fetal bovine serum (Gibco®) and cultured at 37 °C in a humidified incubator with 5% CO_2_, as indicated by the vendor. After reaching the desired confluency, the cells were detached using Accutase (StemPro®) and quantified via the trypan blue method. Before cell seeding, the micro needle substrate was first sterilized in 100% ethanol for an hour, then thoroughly washed with phosphate buffered saline (PBS) and cell medium. The cells were allowed to grow for 24 hours before all experiments were performed.

### Cytotoxicity and intracellular delivery assessment

The cytotoxicity of the micro needles was evaluated using the LIVE/DEAD Viability/Cytotoxicity Kit (Molecular Probes™), following vendor indications. Briefly, the cells were washed with PBS and then stained with a 2 µM calcein AM/4 µM ethidium homodimer-1 (EthD-1) solution for 35 minutes at room temperature. The cells were then washed with PBS and imaged using a Leica DMI6000 B fluorescence microscope.

Similarly, double staining of cells with calcein and EthD-1 was used to quantify intracellular access with the micro needles. The calcein AM/EthD-1 dye combination works through exclusion: while the green dye calcein AM passes through the cell membrane and stains live cells, the red dye EthD-1 cannot access the cell cytoplasm unless the cell membrane is compromised (i.e. as in cell death). Therefore, the co-localization of the two dyes can only be expected if the cell is alive and EthD-1 has been transported inside the cells.

For the magnetic field experiments with biological samples, the micro needle substrate with live cells was positioned in a 35 mm cell culture dish (Nunclon®) in PBS and placed in the middle of the coil, as described previously.

### Scanning electron microscopy imaging

The cells cultured on the micro needles were fixed in 2.5% glutaraldehyde in 0.1 M cacodylate buffer for 2 hours at room temperature. The cells were then washed with cacodylate buffer and then post-fixed in 1% osmium tetroxide (Electron Microscopy Sciences) in the same buffer for 1 hour in the dark. Following a thorough wash with deionized water, a serial dehydration of the sample was performed using ethanol at increasing concentrations, starting at 10% and ending at 100% purity. The sample was then dried at the critical point (Automegasamdri®-915B) in 100% ethanol before imaging under a scanning electron microscope (SEM, Quanta 600. FEI Company).

### TEM preparation

The cells were fixed with 2.5% glutaraldehyde in cacodylate buffer (0.1 M, pH 7.4) for 48 h.

Osmication was performed using reduced osmium (1:1 mixture of 2% osmium tetroxide and 3% potassium ferrocyanide).

The samples were dehydrated in ethanol series and flat embedded in epoxy resin. After polymerization, the lift off was done using liquid nitrogen. Thin sections (80 nm to 120 nm) were collected on copper grids and contrasted with lead citrate.

Imaging was performed using a transmission electron microscope (TEM) operating at 300 kV (Titan Cryo Twin, FEI Company, Hillsboro, OR). Images were recorded on a 4k × 4k CCD camera (Gatan Inc., Pleasanton, CA).

## Electronic supplementary material

Supplementary Material
