# Supplementary material for: Inductively actuated micro needles for on-demand intracellular delivery
Source: Sci Rep. 2018 Jul 2;8:9918. doi: 10.1038/s41598-018-28194-3 (PMC6028653; doi:10.1038/s41598-018-28194-3)
Supplement: Supplementary file 1 — Supplementary Material [file 41598_2018_28194_MOESM1_ESM.pdf]

Supplementary Information

Inductively actuated micro needles for on-demand intracellular delivery

Mincho N. Kavaldzhiev[1,], Jose E. Perez[2], Rachid Sougrat[3], Ptissam Bergam[3], Timothy Ravasi[2], and Jürgen Kosel[1]*

1: Computer, Electrical and Mathematical Science and Engineering
2: Biological and Environmental Sciences and Engineering
3: Imaging and Characterization Core Lab-EM Core lab
King Abdullah University of Science and Technology, Thuwal 23955-6900, Kingdom of Saudi Arabia

4700 King Abdullah University of Science and Technology, Thuwal 23955-6900, Kingdom of Saudi Arabia

Corresponding author email: jurgen.kosel@kaust.edu.sa

Phone number: +966 544700056

Gold needle fabrication and measurements

A four-electrode plating station (SFTinc, model: SEMICON 1500) loaded with gold bath (Elevate Gold 7990, IEW Technic inc) is used for electrodeposition of the needles into the ASi template. The gold bath temperature is maintained at 50 °C with a pH value of 9.5. The deposition is achieved by applying an asymmetric current pulse of amplitudes -1A for 20 ms and 200mA for 300 ms (measured relative to a gold reference electrode) for a total deposition time of 1 min and 30 sec (Figure S1). In order to study the effect of the electrodepositon current density from 3 to 40 mA/cm^2 , the template area is varied, while keeping the same asymmetric current pulse. After deposition, the sample is dismounted from the plating station and washed in DI water. This is done 12 times with a quick dump rinse bath, after which the needles are released from the ASi template. To this end, a XeF_2 vapor etch system (Xactic X3

system) is used to remove the amorphous silicon leaving free standing gold needles on top of the gold electrode. Finally, a reactive ion etch step (ICP 1500, Ar 30sccm, 10mTorr) is applied for 2 min and 30 sec to remove the bottom gold electrode.

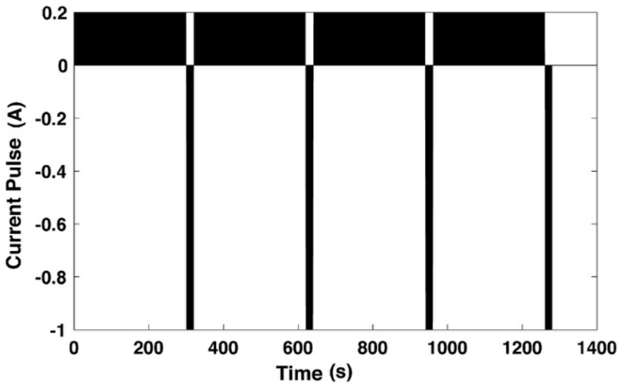

Figure S1 : Forward and reverse current pulses in a rectangular current wave form applied for the electrodeposition of gold (Elevate 7990).

Gold is used as the material for the micro needles, because of its low electrical resistance, electrochemical properties, chemical stability and wide spread in-vivo and in-vitro applications. However, the process can be applied to fabricate needles made of other materials, such as platinum, nickel, cobalt, iron with the same four-electrode plating station and ASi template.

Induction heating setup and temperature measurements

A magnetic inductive heating system (IEW Induktive Erwarmungsanlagen GmbH, TTH3) is used to generate an alternating magnetic field with a frequency of 425 kHz. A five turn water cooled copper coil with 3.8 cm inner radius, 5.2 cm outer radius and 5.5 cm height is installed as a field source (Figure S2 (a),(b)). Additional cooling is performed using a brushless motor fan (ME80251V1, SUNON) positioned 15 cm away from the coil and supplied with 5 ± 0.25 V (Agilent E3631A, DC power supply). The sample is positioned in the middle of a glass bottle (Pyrex 10mL, Corning) with 1 mL of DI water, which is placed in the middle of the coil.

The temperature is measured with a fiber optic probe (STM2-S01821, Lumasense Technologies) installed on a thermometer (Luxtron 812, Lumasense Technologies), manually positioned at close proximity to the surface of the sample and completely submerged in DI water.

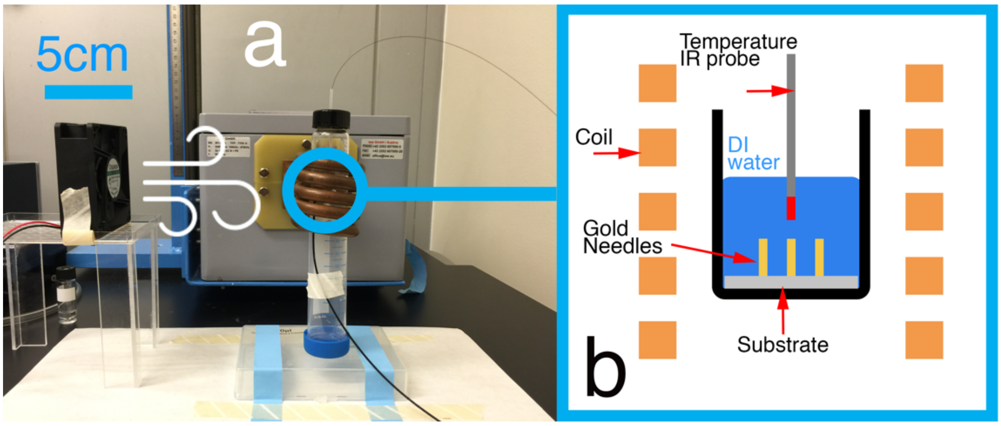

Figure S2 : a) Electromagnetic induction heating setup with the sample, fan and coil. b) Schematics of the infrared (IR) temperature probe in proximity to the sample with gold needles.

Inductive field heating experiments were conducted with magnetic fields of 425 kHz and an amplitude of 90 Oe (Figure S3 black curve) or 360 Oe (Figure S3 red curve). The sample subjected to inductive heating was composed of 43200 gold needles covering a 1 cm^2 area with filled needles of 1.5 μm diameter and 5 μm height. The inductively heated micro needles heated the 1 mL Di water from room temperature to 36°C at 90 Oe and 45°C at 360 Oe after 10 min as seen in Figure S3. The system (DI water and needles) does not reach thermal equilibrium for the time of the measurement (15 min). However, after about 5 min the temperature increase slows down considerably. As a control experiment, the temperature of 1 mL of DI water without needles is also shown. The temperature increases by 3°C after 15 min at 360 Oe, which is due to external heat radiated from the induction coil, and confirms the inductive field heating of the gold needles.

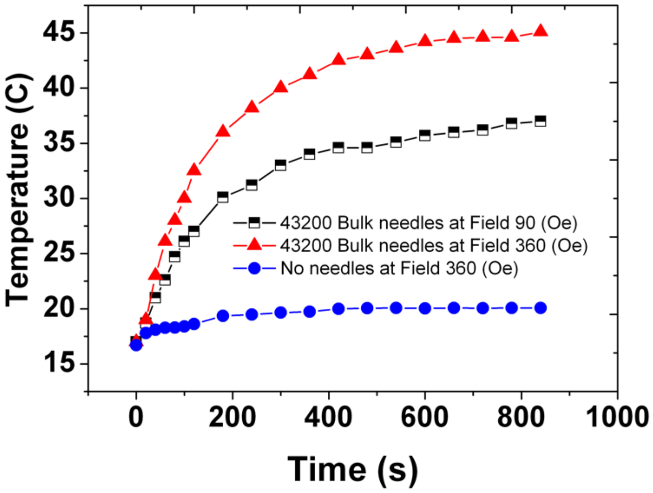

Figure S3 : Inductive heating of an array of 43200 gold needles submerged in 1 ml of DI water with a magnetic field of 425 kHz and two different amplitudes compared to a reference substrate without gold micro needles.

Template and Electrolyte characterization

An SEM image of the ASi template's cross-section with a gold electrode is presented in Figure S4a. The Cr hard mask is preserved and not affected by the DRIE process applied to create the holes. The selectivity of the DRIE also prevents affecting the 100 nm thick gold electrode layer. An overgrowth of gold can be observed on top of the template as rings around the holes (Figure S4b). These gold rings can be removed by applying 1 min of reactive ion etching without affecting the Cr hard mask significantly.

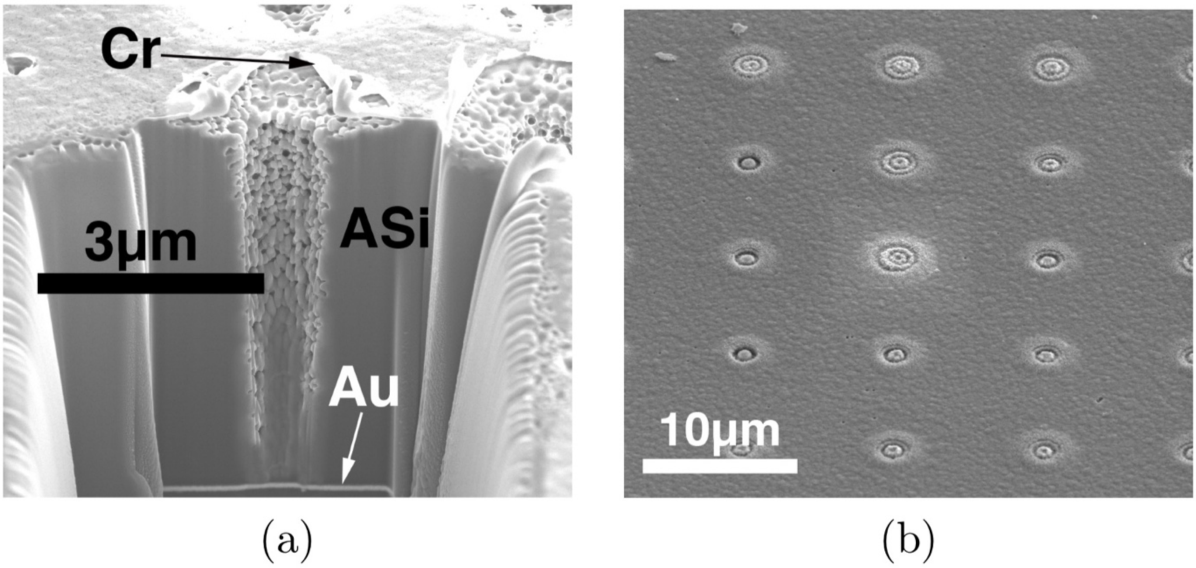

Figure S4 : SEM images of the amorphous Si template. a) Cross sectional image of an ASi template after etching, showing the gold electrode layer and protective layer of Cr. b) An ASi template after the gold electroplating.

A cyclic voltammetry study of the gold electrolyte has been performed according to standards set by[4], where a range of voltages is applied to characterize the solution's electroactivity. The characterization took place in a commercial potentiostat (PGSTAT204, Metrohm Autolab) with a silver reference electrode (Ag-AgCl/3M KCI, Metrohm 6.0733 Autolab) and working/counter platinum electrodes (0.9 cm/1.6 cm/0.1 cm). The gold electrolyte was subjected to a range of triangular potential wave functions (Figure S5a), giving rise to a current-voltage electrolyte characteristic curve.

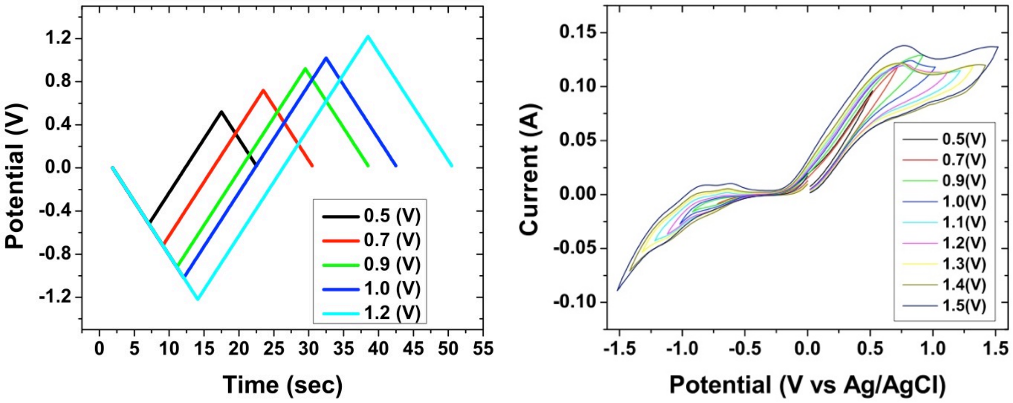

(a) (b)

Figure S5 : a) Cyclic voltammetry potential functions applied in a standard oxidation and reduction reaction occurring during the gold electrolyte measurements. The scans are in a range between 0.5V and 1.5V. b) Current-voltage diagrams corresponding to the potential

The gold electrolyte solution has been maintained at 50°C (gold needle temperature deposition), constant concentration and a pH of 9.5. A gold electrolyte with such pH value is characteristic for cyanide-based electrolytes. The gold electrolyte exhibits a standard current/voltage behavior[5] (Figure S5b) with a cathodic current peak of 12.5 mA for a voltage of 1 V.-The electrolyte turns black with a voltage greater than 1.2 V, due to hydrolysis.

Additional cell control studies

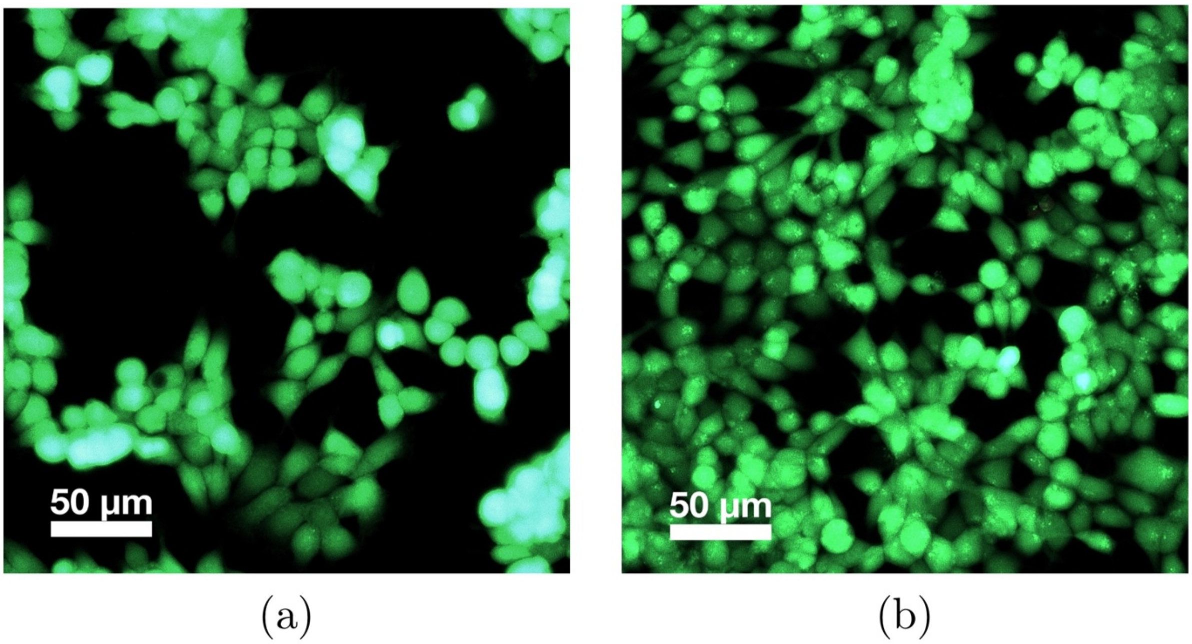

Figure S6 : Fluorescence microscopy of calcein/EthD-1 stained HCT116 cells (a) HCT116 cells on SiO2 surface without needles after 5 min of magnetic field application and (b) 24 h post-treatment, respectively.

We studied the effects of an alternating magnetic field on the cells. For this purpose, cells were grown on a silicon template without gold micro needles and a field of 360 Oe and 425 kHz was applied for five minutes. The results immediately after the field application (Figure S6a) and 24 hours later (Figure S6b) confirm that cell growth and viability are not affected by the magnetic field.

References :

1. Karni, M., Zidon, D., Polak, P., Zalevsky, Z. & Shefi, O. Thermal degradation of DNA. *DNA Cell Biol.* **32,** 298–301 (2013).
2. Nagy, A. Thermal stability of chemically denatured green fluorescent protein (GFP): A preliminary study. *Thermochimica Acta* **410,** 161–163 (2004).
3. Wang, X., Lim, H. J. & Son, A. Characterization of denaturation and renaturation of DNA for DNA hybridization. *Environmental Health and Toxicology* **29,** e2014007 (2014).
4. Kohl, P. A. in *modern electroplating* 1–16 (2016).
5. Elias, J. *et al.* Electrodeposition of gold thin films with controlled morphologies and their applications in electrocatalysis and SERS. *Nanotechnology* **23,** (2012).
